# ERBB2 Increases Metastatic Potentials Specifically in Androgen-Insensitive Prostate Cancer Cells

**DOI:** 10.1371/journal.pone.0099525

**Published:** 2014-06-17

**Authors:** Jessica Tome-Garcia, Dan Li, Seda Ghazaryan, Limin Shu, Lizhao Wu

**Affiliations:** 1 Rutgers New Jersey Medical School-Cancer Center, Newark, New Jersey, United States of America; 2 Department of Microbiology and Molecular Genetics, Rutgers New Jersey Medical School, Newark, New Jersey, United States of America; Peking University Health Science Center, China

## Abstract

Despite all the blood-based biomarkers used to monitor prostate cancer patients, prostate cancer remains as the second common cause of cancer mortality in men in the United States. This is largely due to a lack of understanding of the molecular pathways that are responsible for the aggressive forms of prostate cancers, the castrate-resistant prostate cancer and the metastatic prostate cancer. Cell signaling pathways activated by the *ERBB2* oncogene or the *RAS* oncogene are frequently found to be altered in metastatic prostate cancers. To evaluate and define the role of the ERBB2/RAS pathway in prostate cancer metastasis, we have evaluated the impact of *ERBB2*- or *RAS*-overexpression on the metastatic potentials for four prostate cancer cell lines derived from tumors with different androgen sensitivities. To do so, we transfected the human DU145, LnCaP, and PC3 prostate cancer cells and the murine Myc-CaP prostate cancer cells with the activated form of *ERBB2* or *H-RAS* and assessed their metastatic potentials by three complementary assays, a wound healing assay, a transwell motility assay, and a transwell invasion assay. We showed that while overexpression of *ERBB2* increased the metastatic potential of the androgen-insensitive prostate cancer cells (i.e. PC3 and DU145), it did not affect metastatic potentials of the androgen-sensitive prostate cancer cells (i.e. LnCaP and Myc-CaP). In contrast, overexpression of *H-RAS* only increased the cell motility of Myc-CaP cells, which overexpress the human *c-MYC* oncogene. Our data suggest that ERBB2 collaborates with androgen signaling to promote prostate cancer metastasis, and that although RAS is one of the critical downstream effectors of ERBB2, it does not phenocopy ERBB2 for its impact on the metastatic potentials of prostate cancer cell lines.

## Introduction

Prostate cancer is the most common non-cutaneous cancer and the second leading cause of cancer mortality in men in the United States [1]. Despite increased screening for early detection and monitoring, prostate cancer-specific mortality has remained at the same level [2]. This is likely due to both the inability to diagnostically distinguish between the non-invasive, indolent localized prostate cancers and the very aggressive localized cancers with high metastatic potentials, and the poor understanding of the cellular and molecular basis for metastatic prostate cancers [3].

One of the best studied genes in human malignancies, including prostate cancer, is the *ERBB2* or *HER2* or *NEU* oncogene. ERBB2 is a member of the epidermal growth factor receptor (EGFR) family, which consists of four members (EGFR, ERBB2, ERBB3 and ERBB4) that act as tyrosine kinase receptors [4]–[7]. They are considered as potent mediators of cell growth and cancer development [8]–[10]. In breast cancer, amplification or overexpression of *ERBB2* is a common event that appears in 15–30% of all specimens [11], and *ERBB2* gene amplification and/or overexpression have been associated with a poor clinical outcome [12], [13]. Consistent with an important role of ERBB2 in breast cancer metastasis, overexpression of a constitutively activated form of *ERBB2* (i.e. *NeuT*) [14] in mice is sufficient to trigger metastatic mammary tumors [15]. However, the potential role of ERBB2 in the development of metastatic prostate cancer is unclear partly because various attempts to assess frequencies of *ERBB2* amplification/overexpression in human prostate cancer samples yielded inconsistent results [16]–[25]. Interestingly, *ERBB2* overexpression has been implicated in androgen-resistant metastatic prostate cancers [26], suggesting a possible role for ERBB2 in the acquisition of metastatic potentials of prostate cancer cells.

Overexpression of *ERBB2* results in the induction of several signaling pathways, such as the phosphoinositide-3-kinase/protein kinase B (PI3K/AKT) pathway and the mitogen-activated protein kinase (MAPK) pathway [27]. Both the PI3K/AKT pathway and the MAPK pathway regulate cellular proliferation and cell survival, and have been implicated in cancer metastasis [28]–[30]. The principal downstream effector of ERBB2 that regulates these two kinase pathways is the oncogenic *RAS*, although ERBB2 is also able to activate PI3K/AKT independent of the *RAS* activation [31]. Importantly, PI3K/AKT and MAPK are the only RAS-effector pathways commonly mutated in human cancers [32].

*RAS* oncogenes encode three monomeric GTPases, H-RAS, N-RAS, and K-RAS, which are activated when bound to GTP. While inhibition of *RAS* in androgen-independent PC3 prostate cancer cells and androgen-dependent LnCaP prostate cancer cells led to growth arrest and apoptosis [33], constitutive activation of the RAS/MAPK pathway in LnCaP prostate cancer cells promoted androgen hypersensitivity [34]. In addition, immunohistochemical analysis of hormone-sensitive and hormone-refractory prostate cancer specimens showed that increased expression of *N-RAS* was associated with hormone-refractory prostate cancers, and was correlated with shorter time to tumor relapse and reduced disease-specific survival [35]. In a xenograph mouse model, activation of two RAS effector pathways, *Raf/ERK* and *RalGEF*, in the moderately metastatic DU145 prostate cancer cell line promoted metastasis to the brain and bone, respectively [36]. These data suggest a possible role of RAS in promoting metastasis in human prostate cancers.

To further define the potential roles of the ERBB2/RAS pathway in promoting prostate cancer metastasis, we have examined the effects of overexpression of *ERBB2* or *RAS* on the metastatic properties of three human prostate cancer cell lines and one murine prostate cancer cell line with various levels of androgen sensitivities and different metastatic potentials. To do so, we first transfected three commonly used human prostate cancer cell lines (DU145, LnCaP, and PC3) and one murine prostate cancer cell line (Myc-CaP) with the activated form of *ERBB2* or *H-RAS*. We then evaluated the metastatic potentials of the genetically modified cells by three different complementary assays, a wound healing assay, a transwell motility assay, and an invasion assay. We found that while overexpression of *ERBB2* increased metastatic potentials specifically for androgen-insensitive human prostate cancer cells, overexpression of *RAS* did not have similar impacts on metastatic potentials but specifically increased cell motility of *c-MYC*-overexpressing murine Myc-CaP cells.

## Methods

### Cell Lines and Cell Culture

Myc-Cap is a non-metastatic, androgen-sensitive murine prostate cancer cell line that was established from primary prostate tumors isolated from the *Pb-Hi-Myc* mice [37]. LnCaP [38], DU145 [39], and PC3 [40] are three human metastatic prostate cancer cell lines with different androgen sensitivities and different metastatic properties (Table 1). LnCaP and PC3 cell lines were maintained in RPMI 1640 medium, and Myc-CaP cells were grown in DMEM. Both media were supplemented with 10% fetal bovine serum (FBS). DU145 cells were maintained in DMEM:Ham’s F12 medium (1∶1) supplemented with 10% newborn calf serum. Amphotropic Phoenix cells were used for retroviral transfection and were maintained in DMEM supplemented with 12.5% FBS. Senescent BJ human skin fibroblast cells were generated by replicative senescence [41] and were used as a positive control for β-galactosidase activity assay. They were maintained in DMEM supplemented with 10% FBS. All cells were cultured in a humidified incubator at 37°C with 5% CO_2_.

**Table 1:** Summary of the principal characteristics of the prostate cancer cell lines included in the study.

Cell line	Origin	Metastasis level	Androgen sensitivity	References
PC3	Human bone metastasis	High	Insensitive	[40], [61], [62]
DU145	Human brain metastasis	Moderate	Insensitive	[39], [61], [62]
LnCaP	Human lymph node metastasis	Low	Sensitive	[38], [61], [62]
Myc-CaP	Mouse primary prostatecarcinoma	Non-metastatic	Sensitive	[37]

### Retroviral Transfection

Retroviral overexpression vectors *pBabe-Puromycin-H-Ras* and *pBabe-Puromycin-ERBB2*, which overexpress a mutated form of human *H-RAS* gene (*H-RAS^G12V^*) and a constitutive activated form of human *ERBB2* gene (*NeuT*), respectively, were gifts from Dr. Gustavo Leone. High-titer viruses were produced by calcium phosphate transient transfection of retroviral constructs into amphotropic Phoenix packaging cells as previously described [42]. We infected prostate cancer cells with fresh retroviruses using standard methods in the presence of polybrene (4 µg/ml). Infected cells were subjected to selection with puromycin (2.5 µg/ml) for five days. Puromycin-resistant cells were cultured in fresh DMEM without puromycin for one day before being either harvested to prepare cell lysates for Western blotting analysis, or re-plated for experiments. All data presented were collected by using cells from at least two independent retroviral transfections that yielded similar levels of *ERBB2* and *RAS* overexpression.

### Western Blot

Cell lysates with equal amounts of proteins (30 µg) were separated in 8% SDS-PAGE, except for ERK and pERK, for which cell lysates were separated in 10% SDS-PAGE. Separated proteins were then electrophoretically transferred to a 0.45 µM nitrocellulose membrane (GE Healthcare, Buckinghamshire, UK), which was subsequently blocked at 4°C for 1 hr with 5% nonfat dry milk in TBST (50 mM Tris, pH 7.5, 0.15 M NaCl, 0.1% Tween 20 (w/v)). The blots were then incubated with appropriate dilutions of primary antibodies overnight at 4°C in TBST containing 3% nonfat dry milk. Primary antibodies used for Western blot analysis include polyclonal antibodies for H-RAS (sc-520), E2F1 (sc-193), E2F2 (sc-633), ERK1/2 (sc-135900), and p-ERK1/2 (sc-81492) from Santa Cruz Biotechnology (Dallas, TX); actin (A2066) from Sigma (St Louis, MO); ERBB2 (MS-730-PI) from Thermo Fisher Scientific (Fremont, CA); AKT (4691S), p-AKT (4060S), p38 (9212S), and p-p38 (9211S) from Cell Signaling (Beverly, MA). After washing 10 min for three times in TBST, the blots were incubated with horseradish peroxidase-conjugated secondary antibodies from Perkin Elmer (Boston, MA) either against rabbit (NEF 812001EA) or against mouse (NEF 822001EA) with a dilution of 1∶3000 in TBST with 3% milk. After three washes with TBST for 10 min each, the blots were incubated at room temperature for 1 hr with ECL from Thermo Fisher Scientific (Rockford, IL), and exposed to an X-ray film for autoradiography. Antibodies against actin were used as loading controls. Quantification of the protein levels based on band intensities on Western blots was carried out with the Image J software (NIH, MD, USA, http://rsb.info.nih.gov/ij/).

### Wound Healing Assay

The migration ability of the cells was evaluated by using a wound healing assay as previously described with slight modifications [43]. Cells were plated in 35 mm dishes and left to grow until reaching 100% confluence. Confluent cells were maintained under the same culture conditions for 48 hr to induce density arrest and to minimize cellular proliferation. Confluent plates were scratched down three times with a 200 µl pipette tip, creating left, center, and right scratches/wounds that crossed with two horizontal lines previously drawn as landmarks for quantifications. After the scratching, plates were washed once with medium to remove floating cells, and were replaced with fresh medium. Cells were allowed to migrate across the wounds and pictures were taken at various time points using a phase-contrast microscope until the wound was completely closed. The time points at which the pictures were taken depended on the migratory ability of different cell lines studied. Each experiment was performed in triplicates and was repeated at least once to validate the initial data.

### Motility Assay (Boyden Chamber Assay)

The motility of the cells was also evaluated using cell culture inserts from BD Falcon (Franklin Lakes, NJ), and following the protocol previously established with minor modifications [44]. Cells that were previously maintained in a starvation medium with 0.2% FBS for 24 hr to minimize cellular proliferation, were resuspended at a concentration of 2×10^5^ cells/ml in medium containing 0.2% FBS. 1×10^5^ cells or 500 µl of cell suspensions were plated onto each insert, which was previously coated with 3 µg/ml of rat tail collagen solution from BD (Bedford, MA) overnight at room temperature. The lower chamber contained medium with 10% FBS as a chemo-attractant. Cells were allowed to pass through the porous membrane and were collected at different time points that were empirically determined based on the migratory ability of each cell line. Non-migratory cells were then removed from the surface of the membranes using cotton swabs. Cells that passed through the pores of the membrane were fixed and stained using the Diff-Quick nuclei and cytoplasm staining kit (Dade Behring, Newark, DE). Stained migratory cells were counted under a microscope. Each experiment was performed in triplicates and was repeated at least once to validate the initial data.

### Invasion Assay

The invasiveness of the cells was measured using cell culture inserts from BD Falcon (Franklin Lakes, NJ) by following a previously described protocol with minor modifications [45]. As in the transwell-based motility assay described above, cells were maintained in a starvation medium with 0.2% FBS for 24 hr before seeding to minimize cellular proliferation. Inserts were coated with 50 µg/ml of rat tail collagen solution from BD (Bedford, MA) for 5 hr at room temperature. After the incubation, inserts were washed three times with serum-free medium and were allowed to dry at 37°C overnight. Once dried, membranes were covered with 100 µl of collagen solution at a final concentration of 1.3 mg/ml (for all cells) or with 100 µl of Matrigel (Cat. #356231) from BD (Bedford, MA) at a final concentration of 300 µg/ml (for Myc-CaP cells only), and were allowed to solidify at 37°C. Cells were resuspended at a concentration of 2×10^5^ cells/ml in medium containing 0.2% FBS. 1×10^5^ cells or 500 µl of cell suspensions were plated onto each insert. The remaining procedure was performed as described above in the motility assay section. Each experiment was performed in triplicates and was repeated at least once to validate the initial data.

### Assessment of Senescence-associated β-galactosidase Activity

The endogenous β-galactosidase activity of prostate cancer cells was assessed by X-gal staining as previously described [46]. Cells seeded in 35 mm dishes in triplicates were fixed for three min at room temperature in 1X PBS containing, 2% formaldehyde and 0.2% glutaraldehyde. After two consecutive washes with 1X PBS, cells were incubated for 16 hr at 37°C with staining solution (2 ml per dish) that consists of X-gal at a final concentration of 1 mg/ml in dimethylformamide, 40 mM citric acid/sodium phosphate buffer pH 5.7 (0.1 M citric acid/0.2 M sodium phosphate), 5 mM potassium ferrocyanide, 5 mM potassium ferricyanide, 150 mM sodium chloride, and 2 mM magnesium chloride. Pictures were taken under a phase-contrast microscope. Positive and negative cells were counted from at least three different fields.

### Cell Growth Rate Assessment

Prostate cancer cells were seeded in 6-well plates in triplicates. The density of the initial seeding was empirically determined to allow us to count at least four time points before cells reached 100% confluence. Thus, cells were seeded as follows: 70000 cells for PC3, 35000 cells for LnCaP, 120000 cells for DU145, and 70000 cells for Myc-CaP. Twelve hours after seeding, cells were counted using a hemocytometer (Hausser Scientific, Horsham, PA) to be normalized as the corresponding seeding cell number and to be used as the first time point. Cells were then counted every 12 hr for PC3 and Myc-CaP cell lines or every 24 hr for DU145 and LnCaP cell lines. Growth rates were estimated by calculating and comparing the linear slope for each growth curve.

### Statistical Analysis

Values are presented as mean ± SD. Statistical significance was determined by Student’s *t*-test with a significance threshold of *P*<0.05. In all figures, statistical significances were denoted as ^*^ *P*<0.05, **P<0.01, and ***P<0.001.

## Results

### Overexpression of *ERBB2* and *RAS* Oncogenes in Prostate Cancer Cell Lines by Retroviral Infection

**Figure 1:**
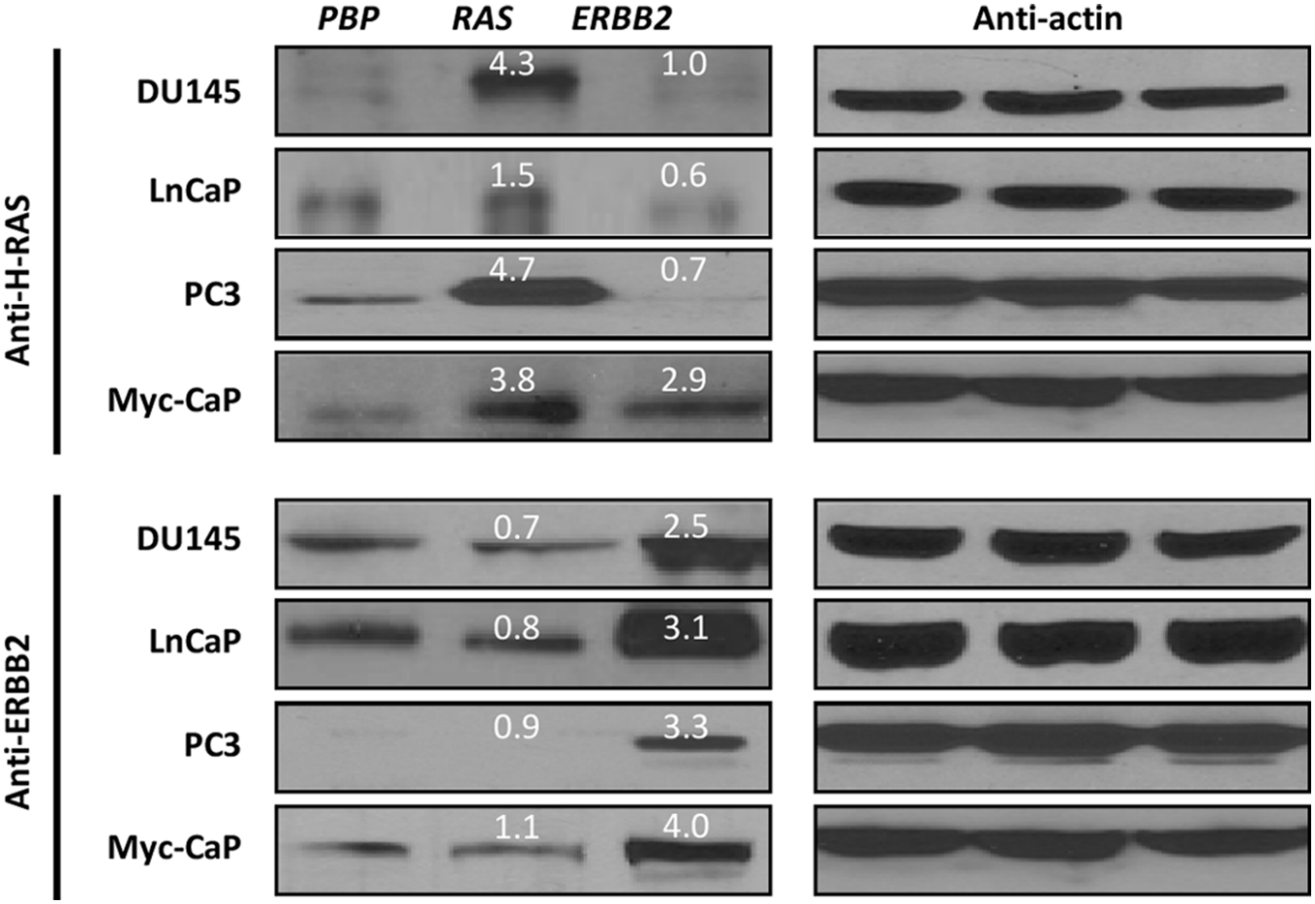
Moderate overexpression of *RAS* and *ERBB2* by retroviral transfections of prostate cancer cells. ERBB2 and RAS protein levels were assessed by Western blots using antibodies against H-RAS or ERBB2 for total cell lysates prepared from prostate cancer cells transfected with either control retroviruses (*PBP*), or retroviruses overexpressing *PBP*-*H-RAS* (*RAS*) or *PBP-ERBB2* (*ERBB2*). Blots with antibodies against actin served as loading controls. Numbers in white represent fold changes in ERBB2 or RAS protein levels in *ERBB2*- or *RAS*-overexpressing cells relative to those in their corresponding *PBP* control cells after the actin normalization.

To overexpress *ERBB2* and *RAS* oncogenes in prostate cancer cell lines, we transfected prostate cancer cells with *pBabe-Puromycin-* (*PBP-*) based retroviruses overexpressing an activated form of *ERBB2* (*PBP-ERBB2*) or a mutated form of *H-RAS* (*PBP-RAS*). As shown in Figure 1, Western blotting analysis indicated that transfection of cells with *PBP-RAS* retroviruses led to moderate up-regulations of *RAS* ranging from 1.5 fold (for LnCaP) to 4.7 fold (for PC3), and that transfection of cells with *PBP-ERBB2* retroviruses led to moderate up-regulations of *ERBB2* ranging from 2.5 fold (for DU145) to 4.0 fold (for Myc-CaP) Interestingly, although *RAS* overexpression did not change protein levels of ERBB2, *ERBB2* overexpression elevated protein levels of RAS (2.9 fold) specifically in the Myc-CaP cells (Figure 1), which overexpresses the human *c-MYC* oncogene [37].

### Overexpression of *ERBB2* Leads to Moderate Increases in Cell Growth in LnCaP, DU145 and PC3 Cells

**Figure 2:**
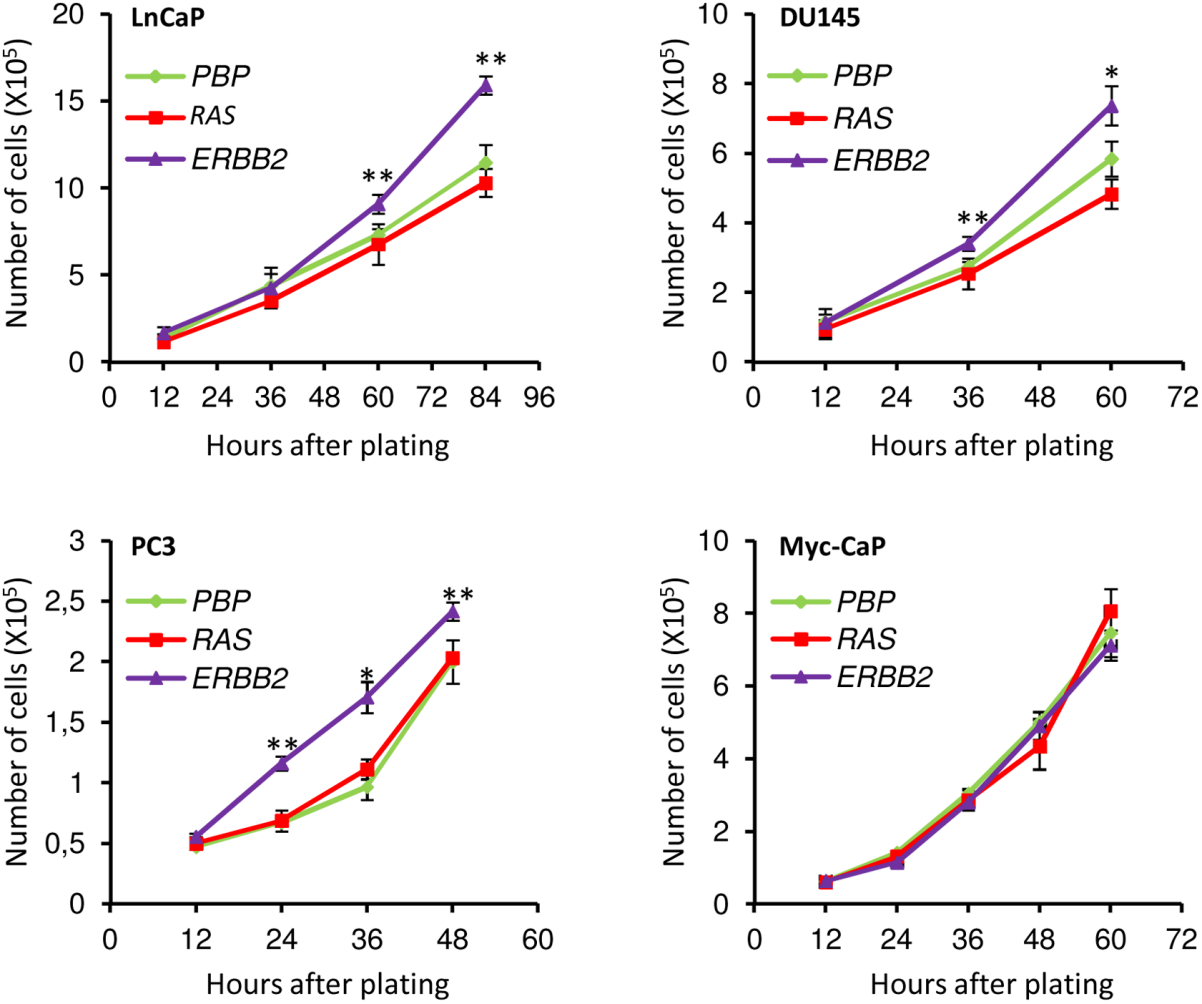
Overexpression of *ERBB2* led to moderate increases in cell growth of human prostate cancer cells. Cell growth rates were assessed by cell counting every 12 hours or 24 hours for various prostate cancer cells that were transfected with control retroviruses (*PBP*), or retroviruses overexpressing either *PBP-H-RAS* (*RAS*) or *PBP-ERBB2* (*ERBB2*).

Considering that activation of the *ERBB2*/*RAS* signaling pathway in prostate cancer cells may affect their growth rates, which could influence the analysis to assess their metastatic potentials, we carried out a growth curve assay using asynchronous cells. As shown in Figure 2, *ERBB2* overexpression led to moderate increases in growth rates for all of the three human prostate cancer cell lines: an average of 43% increase for LnCaP cells, 33% increase for DU145 cells, and 25% increase for PC3 cells. However, overexpression of *ERBB2* did not affect the growth rate of the murine prostate cancer cell line, Myc-CaP. In contrast, *RAS* overexpression did not have significant effects on the growth rates of any of the four cell lines (Figure 2).

### Overexpression of *RAS* Reduces Cell Migration Rates of LnCaP and DU145 Cell Lines

**Figure 3:**
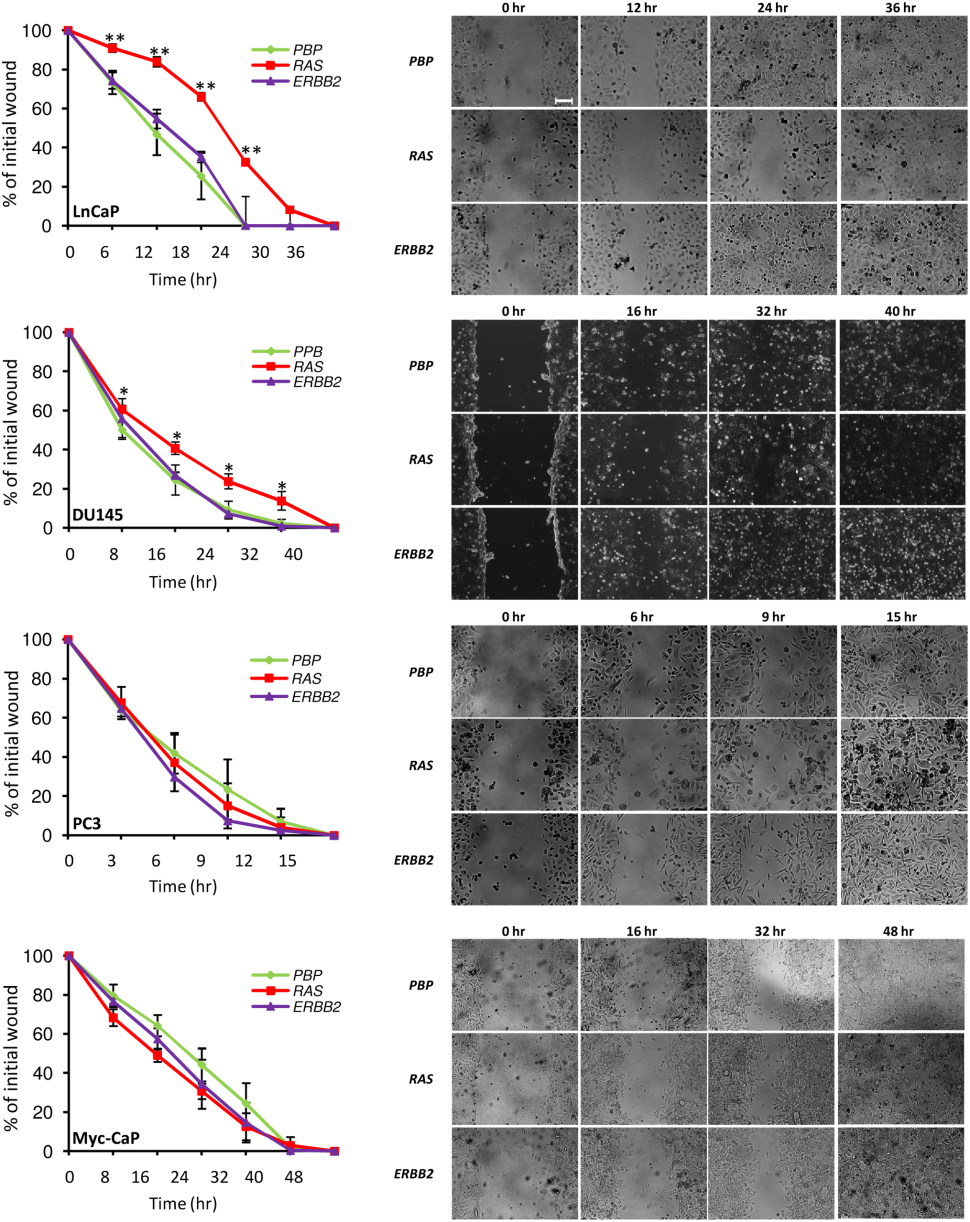
Overexpression of *RAS* or *ERBB2* did not increase lateral cell migration rates. Cell migration rates were estimated by a wound healing assay for prostate cancer cells that were transfected with either control retroviruses (*PBP*), or retroviruses overexpressing *PBP*-*H-RAS* (*RAS*) or *PBP-ERBB2* (*ERBB2*). Left panels showed percentages of wounds remained at different time points. The percentages of wounds were estimated based on the average of 12 measurements on each plate reflecting measurements of four evenly distributed sections on each of the three wounds/scratches on each plate. Data were presented as means ± SD from three replicates. Right panels showed representative images taken at different time points. All images were taken at the same scale with a scale bar of 200 µM displayed in the first image.

The effects of *ERBB2* and *RAS* overexpression on the metastatic potentials of LnCaP, DU145, PC3, and Myc-CaP prostate cancer cell lines were first assessed by performing a wound healing assay. To overcome potential effects of increased cellular proliferation in *ERBB2*-overexpressing cells (Figure 2) on the wound healing assay, we induced cell density arrest by maintaining confluent plates for 48 hours before making scratches/wounds. As shown in Figure 3, overexpression of *ERBB2* and *RAS* showed various effects on the three human prostate cancer cell lines. Specifically, while overexpression of *ERBB2* had no significant impact on the migration rates of all of the three human prostate cancer cell lines, overexpression of *RAS* significantly decreased the migration rates of LnCaP and DU145 cell lines. Compared to the human prostate cancer cell lines, the murine prostate cancer cell line Myc-CaP seemed to have lower migration rates, as evidenced by the fact that they failed to completely close the wound before they started to grow again around the 32 hour time point (data not shown), regardless of the status of *ERBB2*- or *RAS*-overexpression (Figure 3). Nevertheless, *ERBB2*- or *RAS*-overexpressing Myc-CaP cells had a moderate but statistically insignificant increase in migration rates as compared to control cells (Figure 3).

### *ERBB2* Overexpression Increases Cell Motilities of the Androgen-insensitive DU145 and PC3 Cells, and *RAS* Overexpression Increases the Cell Motility of the Myc-CaP Cells

**Figure 4:**
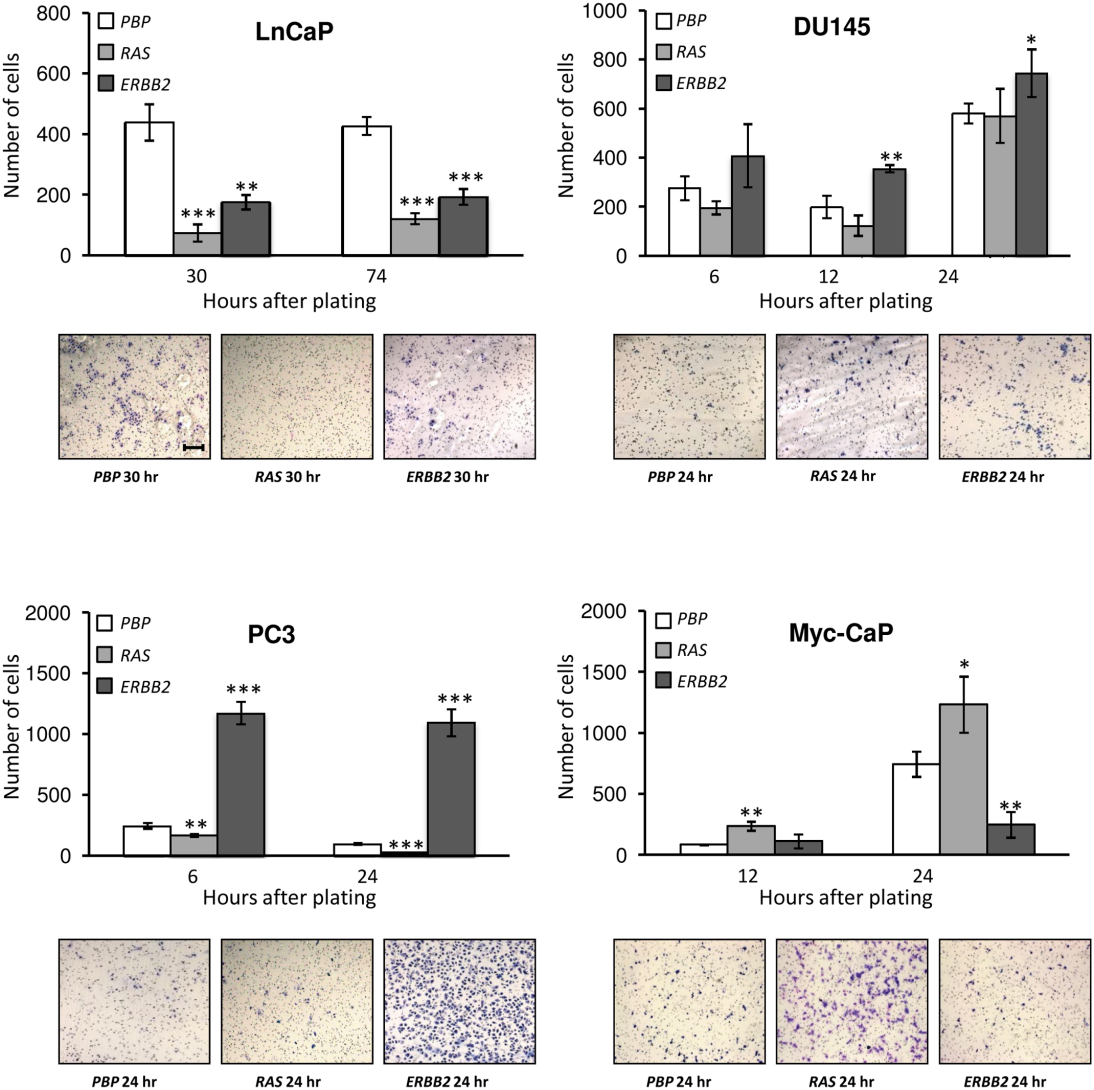
Overexpression of *ERBB2* increased cell motilities of DU145 cells and PC3 cells. Cell motilities were assessed by a transwell-based motility assay for prostate cancer cells that were transfected with either control retroviruses (*PBP*), or retroviruses overexpressing *PBP*-*H-RAS* (*RAS*) or *PBP-ERBB2* (*ERBB2*). Each bar graph showed the relative numbers of cells that have passed through the transwell inserts/membranes, which were stained and counted under a microscope for at least 10 fields per insert. Data were presented as means ± SD from three replicates. Representative images taken at a given time point were shown underneath each bar graph. All images were taken at the same scale with a scale bar of 200 µM displayed in the first image.

To complement the wound healing data presented above, we also assessed the effects of overexpression of *ERBB2* and *RAS* on the cell motility of the prostate cancer cell lines by a transwell-based cell motility assay using porous membrane inserts in transwells. While wound healing assay measures lateral cell motility resulted from the disruption of cell-cell interactions [47], Boyden chambers transwell assay measures chemo-attractive migration, which unlike wound healing assay, is independent of breakages in cell-cell junctions [48]. Therefore, these two assays are complementary and are often used in parallel to gain biological insights on different types of cell migration. To minimize the impact of differential cell growth rates of *ERBB2* overexpression on the motility assay, we induced cell cycle arrest by maintaining cells under serum-starvation conditions for 24 hours before performing the motility assay. As shown in Figure 4, overexpression of *ERBB2* significantly increased cell motilities of the two more metastatic, androgen-insensitive cell lines, DU145 cells and PC3 cells, with a 30% increase and a 6-fold increase, respectively. In contrast, *ERBB2* overexpression significantly reduced the motilities of the two lowly or non-metastatic, androgen-sensitive prostate cancer cell lines, LnCaP and Myc-CaP. In addition, while *RAS* overexpression significantly reduced the motilities of LnCaP cells and PC3 cells, it substantially increased the motility of Myc-CaP cells, which overexpress the *c-MYC* oncogene.

### *ERBB2* Overexpression Promotes Cell Invasiveness in the Androgen-insensitive DU145 and PC3 Cells

**Figure 5:**
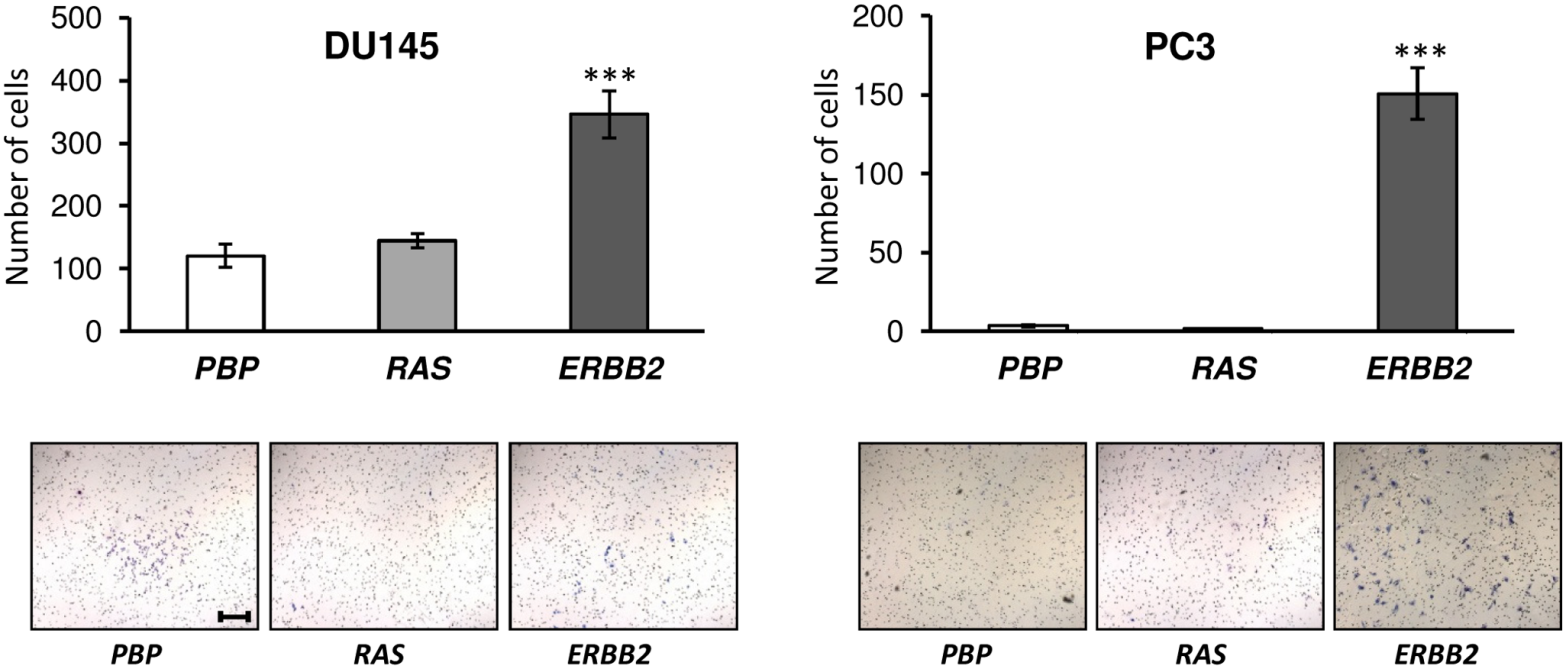
*ERBB2* overexpression increased the invasiveness of DU145 cells and PC3 cells. Cell invasiveness was assessed by a transwell-based invasion assay for prostate cancer cells that were transfected with either control retroviruses (*PBP*), or retroviruses overexpressing *PBP*-*H-RAS* (*RAS*) or *PBP-ERBB2* (*ERBB2*). Each bar graph showed the numbers of cells that have passed through the collagen matrix either 72 hours (for PC3 cells) or 96 hours (for DU145 cells) after plating. Transwell inserts were stained and invading cells were counted for the entire inserts. Data were presented as means ± SD from three replicates. Representative images were shown underneath each bar graph. All images were taken at the same scale with a scale bar of 200 µM displayed in the first image.

The metastatic potentials of *ERBB2*- or *RAS*-overexpression in various prostate cancer cell lines were further assessed by evaluating their relative invasiveness by a transwell-based invasion assay using cell inserts coated with a collagen matrix. This invasion assay allows cells to go through a 100 µM-thick collagen matrix from a low-serum-containing medium to a serum-enriched environment. As shown in Figure 5, the invasiveness of the androgen-insensitive DU145 cells and PC3 cells was substantially augmented in the presence of *ERBB2* overexpression but not in the presence of *RAS* overexpression. These data are consistent with the data from the transwell-based motility assay showing that overexpression of *ERBB2* in DU145 cells and PC3 cells led to increased cell motility (Figure 4). Consistent with their low or no metastatic potentials, neither Myc-CaP cells nor LnCaP cells appeared to be able to penetrate the collagen matrix even after 96 hours of incubation (data not shown). Importantly, overexpression of *ERBB2* or *RAS* was insufficient to enable either Myc-CaP cells or LnCaP cells to pass through the collagen matrix (data not shown).

Since overexpression of *RAS* increased the cell motility specifically in the Myc-CaP cells (Figure 4), we performed a transwell-based invasion assay on Myc-CaP cells using Matrigel to replace collagen. Compared to the collagen matrix, the Matrigel matrix is a reconstituted basement membrane prepared from a mouse sarcoma that better mimics the extracellular environment of a cancer. As in the case of collagen matrix, no cell was seen to pass through the Matrigel matrix either in the control cells or in the *RAS*-overexpressing cells (data not shown).

### Moderate Levels of *RAS* Overexpression does not Promote Cellular Senescence in Prostate Cancer Cells

**Figure 6:**
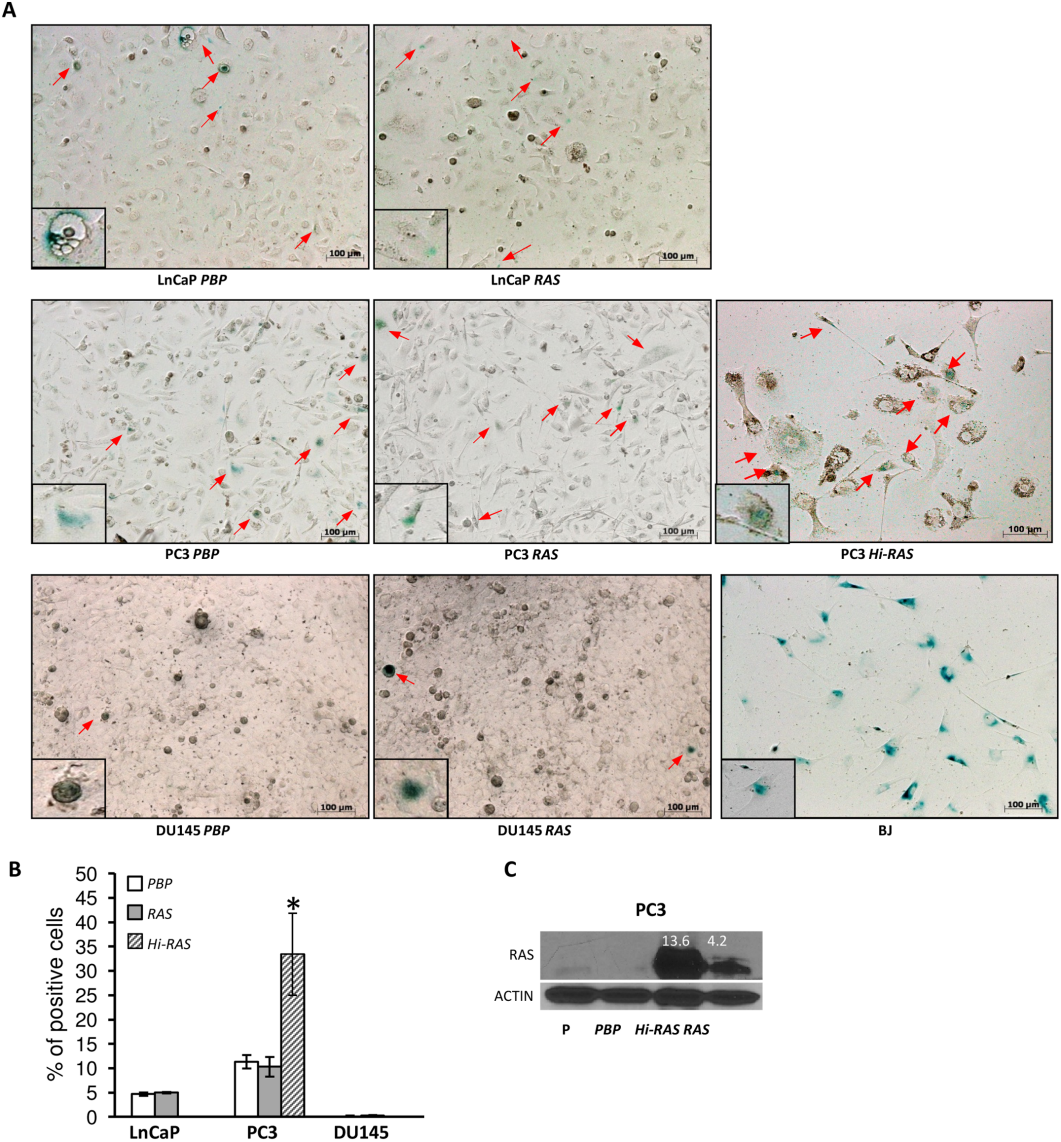
Moderate levels of *RAS* overexpression did not promote cellular senescence in prostate cancer cells. (A) Senescence-associated β-galactosidase activities were assessed by X-gal staining for human prostate cancer cell lines transfected with either control retroviruses (*PBP*) or retroviruses overexpressing *PBP-H-RAS* (*RAS*). PC3 cells expressing an extremely high level of *RAS* (*Hi-RAS*) were included for comparisons. Senescent BJ human skin fibroblast cells were used as a positive control for X-gal staining. Representative images were shown with representative X-gal-positive cells being marked with red arrows. Inserts in each image showed magnified, representative X-gal-positive cells. (**B**) Quantifications of data collected from panel (**A**). Data were presented as means ± SD from three replicates. (**C**) RAS protein levels assessed by Western blot analysis with antibodies against H-RAS for parental PC3 cells (P), PC3 cells transfected with control retroviruses (*PBP*), or PC3 cells transfected with the *PBP-H-RAS* retroviruses overexpressing a moderate level of *RAS* (*RAS*) or a much higher level of *RAS* (*Hi-RAS*). A blot using antibodies against actin was used as a loading control. Numbers in white represent RAS protein levels in fold changes in *ERBB2*- or *RAS*-overexpressing cells relative to those in *PBP* control cells after the actin normalization. Scare bar: 100 µM.

Previous studies have shown that prolonged expression of oncogenic *RAS* in human and rodent primary fibroblast cells *in vitro* [49] or high levels of overexpression of oncognic *RAS* in mammary epithelial cells *in vivo* [50] led to increased cellular senescence. Since *RAS*-overexpressing LnCaP cells and DU145 cells showed reduced abilities to close a provoked wound (Figure 3), and since *RAS*-overexpressing LnCaP cells and PC3 cells showed decreased cell motilities in the transwell mobility assay (Figure 4), we sought to determine whether the decreased mobility in these *RAS*-overexpressing human prostate cancer cells can be explained by a potential increase in cellular senescence in those cells. To this end, we used *in vitro* X-gal staining to assess the senescence-associated β-galactosidase activities, a commonly used biomarker for cellular senescence [51]. As shown in Figure 6A and Figure 6B, moderate levels of *RAS* overexpression in our experimental setting (Figure 1) did not significantly increase cellular senescence in LnCaP, PC3, and DU145 cells, as evidenced by the fact that *RAS*-overexpressing cells showed similar percentages of X-gal-positive cells as their corresponding *PBP* control cells. In contrast, PC3 cells with a much higher level of *RAS* overexpression (i.e. 13.6 fold; Figure 6C, “*Hi-Ras”*) showed a significant increase in the percentage of X-gal-positive cells than either the PC3 cells infected with the control vector (Figure 6C, “*PBP”*) or the PC3 cells with a moderate expression of *RAS* (i.e. 4.2 fold; Figure 6C, “*RAS”*) (Figure 6A and 6B). The increased percentage of X-gal positive PC3 cells with a higher level of *RAS* overexpression is consistent both with their senescence-like morphology (i.e. big and flat) (Figure 6A) and with a previous report that high levels of *Ras* overexpression led to increased cellular senescence [50]. Taken together, our data suggest that the inability of RAS to promote cell motility or invasiveness in human prostate cancer cell lines is not due to premature cellular senescence.

### MAPK and/or PI3K-AKT Pathways are Activated as a Consequence of *ERBB2* or *H-RAS* Overexpression

**Figure 7:**
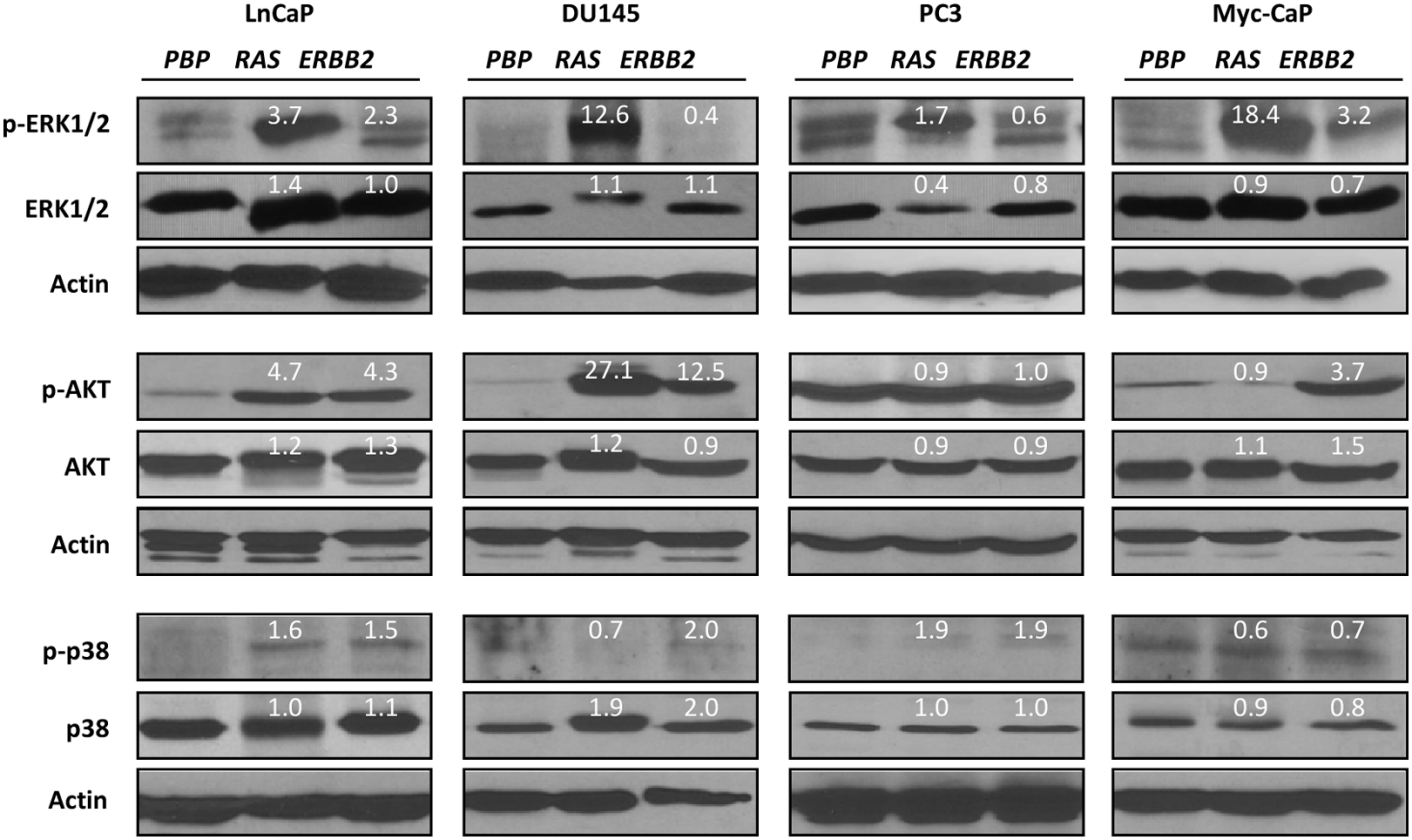
Overexpression of *RAS*/*ERBB2* activates the MAPK pathway and/or the PI3K–AKT pathway in prostate cancer cells. Protein levels for various kinases and their phosphorylated forms were assessed by Western blot analyses for parental prostate cancer cells that were transfected either with control retroviruses (*PBP*), or with retroviruses overexpressing *PBP-H-RAS* (*RAS*) or *PBP-ERBB2* (*ERBB2*). Actin was used as a loading control. Numbers in white represent protein levels in fold changes relative to those in *PBP* control cells after the actin normalization.

The inability of *RAS* overexpression to promote metastatic potentials of prostate cancer cells may be due to a failure to activate its downstream signaling pathways upon moderate *RAS* overexpression. To explore this possibility, we assessed the various downstream effector kinases of the ERBB2/RAS signaling pathway by carrying out Western blot analyses using antibodies against ERK, AKT, and p38 kinases as well as their phosphorylated or activated forms. In parallel, we also carried out the same analysis on *ERBB2*-overexpressing cells. As shown in Figure 7, although overexpression of *ERBB2* or *RAS* did not significantly alter the levels of total ERK, total AKT, or total p38 kinases, they activated the phosphorylated forms of those kinases in most cell lines. Specifically, *RAS* overexpression activates the ERK pathway in all four cell lines as it significantly elevated protein levels of p-ERK, ranging from a 1.7 fold increase in PC3 cells and an 18.4 fold increase in Myc-CaP cells (Figure 7). In addition, overexpression of *RAS* led to significant increases in protein levels of p-AKT in LnCaP cells (by 4.7 fold) and DU145 cells (by 27.1 fold). On the other hand, *ERBB2* overexpression led to significant increases in protein levels of p-ERK in LnCaP cells (by 2.3 fold) and Myc-CaP cells (by 3.2 fold), p-AKT in LnCaP cells (by 4.3 fold), DU145 cells (by 12.5 fold), and Myc-CaP cells (by 3.7 fold), as well as moderate increases in protein levels of p-p38 in LnCaP cells (by 1.5 fold), DU145 cells (by 2.0 fold), and PC3 cells (by 1.9 fold) (Figure 7). Overall, *RAS* overexpression led to the activation of more than one kinase in all cell lines except the Myc-CaP cell line, and *ERBB2* overexpression activated more than one kinase in all cell lines except the PC3 cell line.

## Discussion

Activation of *ERBB2* and *RAS* oncogene is known to trigger cell signaling pathways commonly mutated in human cancers. Therefore, various attempts have been made to determine whether these two oncogenes are involved in the metastatic transformation of prostate cancer cells [36], [52]–[54]. However, up to now, there is no conclusive evidence that links *ERBB2* or *RAS* activation to the invasive cell behavior in prostate tumors. In the present study, we used three complementary approaches to assess the effect of *ERBB2*- and *RAS*-overexpression on the metastatic potentials of three metastatic human prostate cancer cell lines and one non-metastatic mouse prostate cancer cell line that have different androgen-sensitivities. All three human prostate cancer cell lines were derived from metastatic prostate cancers, with PC3 cells being highly metastatic, DU145 cells moderately metastatic, and LnCaP cells lowly metastatic (Table 1). On the other hand, the murine prostate cancer cell line Myc-CaP was derived from non-metastatic primary prostate carcinoma resulted from c-*MYC* overexpression. In addition, while PC3 cells and DU145 cells are androgen insensitive, LnCaP cells and Myc-CaP cells are androgen sensitive (Table 1). We showed that overexpression of *ERBB2* increased metastatic potentials in androgen-insensitive PC3 cells and DU145 cells, as evidenced by increased cell motility and increased invasiveness in those cells. However, *ERBB2* overexpression did not have any significant impact on androgen-sensitive LnCaP cells and Myc-CaP cells. These data suggest that ERBB2 increases metastatic potentials specifically in androgen-sensitive prostate cancer cells.

It has been previously shown that PC3 cells transfected with activated *ERRB2* acquired the potential to metastasize from primary tumor to neighboring soft tissues and skeletons [54], suggesting a potential role of *ERRB2* activation to promote prostate cancer metastasis. In addition, Chung and colleagues showed that one specific single-cell clone (N35) resulted from *ERBB2* overexpression in PC3 cells was found to disseminate widely to the lymph nodes and distant organs upon orthotopic administration [55]. However, subcutaneous administration of the same single-cell clone (N35) and the other *ERBB2*-overexpressing single-cell clone into athymic nude mice did not induce metastasis, suggesting that the ability of ERBB2 to induce prostate cancer metastasis depends on an appropriate host microenvironment [55]. Furthermore, an EGFR tyrosine kinase inhibitor suppressed EGF-induced invasion in hormone-refractory DU145 cell line and PC3 cell line [56]. The fact that *ERBB2* overexpression increased metastatic potentials specifically in the two androgen-insensitive prostate cancer cell lines (but not in the two androgen-sensitive prostate cancer cell lines) suggests that ERBB2 promotes prostate cancer metastasis by collaborating with androgen/androgen receptor signaling. Interestingly, overexpression of *ERBB2* in PC3 cells and DU145 cells led to moderate up-regulations (i.e. a 1.9 fold increase for both cell lines) of activated p38 kinases, an event that is accompanied by moderate down-regulations (i.e. 40% for PC3 cells and 60% for DU145 cells) of activated ERK (Figure 7). It would be interesting to know whether the ability of ERBB2 to increase prostate cancer metastatic potentials depends on the activation of the p38 kinase signaling pathway and the down-regulation of the ERK signaling pathway.

It is worthwhile noting that the two androgen-insensitive prostate cancer cell lines (i.e. PC3 and DU1450) also possess higher metastatic properties than the two androgen-insensitive prostate cancer cell lines (i.e. LnCaP and Myc-CaP) (Table 1). Therefore, our data does not rule out the possibility that the relatively high metastatic property of the PC3 cells and DU145 cells contributes to the ability of ERBB2 to promote prostate cancer metastasis in those cells. It is also worthwhile noting that although overexpression of *ERBB2* increased cell growth in the three human prostate cancer cell lines under growth conditions (Figure 2), such moderate increases in growth rates likely did not make any significant contributions to the ability of ERBB2 to increase metastatic potentials in the PC3 cells and the DU145 cells. This is because all three assays used to assess metastatic potentials were set up under growth arrest conditions, thereby minimizing the potential effects of differential growth rates on our ability to evaluate metastatic potentials. In addition, the moderate increases in growth rates in DU145 cells (a 33% increase) and PC3 cells (a 25% increase) (Figure 2) do not correlate with the much bigger differences in cell motilities and cell invasiveness assessed from the transwell-based motility assay (Figure 4) and the invasion assay (Figure 5). Furthermore, overexpression of *ERBB2* did not increase cell motility or invasiveness in LnCaP cells (Figure 4 and data not shown) despite the fact that it increased growth rate (43%) in LnCaP cells even more than in PC3 cells and DU145 cells (Figure 2).

As one of the critical downstream effectors of ERBB2 pathway, *RAS* oncogene has been previously implicated in prostate cancer metastasis [36]. In the present study, we have shown that overexpression of *H-RAS* and overexpression of *ERBB2* had different impacts on the metastatic potentials of various prostate cancer cell lines. Although overexpression of *ERRB2* led to increased metastatic potentials in PC3 cells and DU145 cells, overexpression of *H-RAS* did not have similar effects on these two cell lines or the LnCaP cell line (Figure 4 and Figure 5), despite the fact that *RAS* overexpression did elevate p-ERK (particularly p-ERK1) as well as p-AKT and/or p-p38 in all of the three human prostate cancer cell lines (Figure 7). These data suggest that *RAS* overexpression does not recapitulate the effect of *ERBB2* overexpression on metastatic potentials of prostate cancer cells and that ERBB2 increases metastatic potentials independent of *H-RAS* activation. Consistent with the latter notion, overexpression of *ERRB2* in the two androgen-insensitive cell lines did not activate *H-RAS* (Figure 1). Interestingly, *RAS* overexpression increased cell motility specifically in the *MYC*-overexpressing Myc-CaP cells (Figure 4), suggesting a collaborative role of MYC and RAS in promoting cell motility. The collaboration between members of the RAS family and c-MYC has been previously established in both mitogenic settings and oncogenic settings. For example, Myc and Ras can collaborate to activate the Rb/E2f pathway to promote cell cycle progression [57]. In addition, conditional activation of *K-ras* and *c-MYC* oncogenes in mice shortened the lymphoma latency compared to inactivation of either oncogene alone, demonstrating that MYC and RAS can collaborate to promote lymphomagenesis [58]. Consistent with a collaborative role of MYC and RAS to promote tumorigenesis, c-MYC-triggered mammary tumorigenesis in mice is preferably coupled with spontaneous *Kras2* mutations [59]. The ability of RAS to collaborate with MYC to activate both mitogenic signaling pathway and oncogenic signaling pathway is likely due to, at least in part, the ability of RAS to stabilize MYC protein [60].

## Conclusions

We have shown that overexpression of the constitutively activated form of *ERBB2* (*NeuT*) increases the metastatic potential of the two androgen-insensitive human prostate cancer cell lines, DU145 and PC3, but not that of the two androgen-sensitive prostate cancer cell lines, LnCaP and Myc-CaP. These findings, coupled with previous xenograph data implicating a potential role of ERBB2 in the promotion of prostate cancer invasiveness or metastasis, strongly suggest a potential crosstalk between the ERBB2 signaling pathway and the androgen/androgen receptor signaling pathway in promoting prostate cancer metastasis. We also showed that overexpression of *H-RAS* in the same four cell lines specifically increased the cell motility of *MYC*-overexpressing Myc-CaP cells, suggesting that MYC collaborates with RAS to promote cell motility.
